# Impaired functional network properties contribute to white matter hyperintensity related cognitive decline in patients with cerebral small vessel disease

**DOI:** 10.1186/s12880-022-00769-7

**Published:** 2022-03-09

**Authors:** Yifan Wang, Xiao Liu, Ying Hu, Zekuan Yu, Tianhao Wu, Junjie Wang, Jie Liu, Jun Liu

**Affiliations:** 1 Department of Radiology, Eye & ENT Hospital of Shanghai Medical School, Fudan University, Shanghai, China; 2 School of Computer and Information Technology, Beijing Jiaotong University, Beijing, China; 3 Institute of Medical Imaging Engineering, School of Medical Instrument and Food Engineering, University of Shanghai for Science and Technology, Shanghai, 200093 China; 4 Academy for Engineering and Technology, Fudan University, Yangpu District, No. 539 Handan Road, Shanghai, 200433 China; 5 Key Laboratory of Industrial Dust Prevention and Control & Occupational Health and Safety, Ministry of Education, Beijing, China; 6 Anhui Province Engineering Laboratory of Occupational Health and Safety, Huainan, China; 7 Laboratory of Industrial Dust Deep Reduction and Occupational Health and Safety of Anhui Higher Education Institutes, Hefei, China; 8 Department of Radiology, Tongren Hospital, Shanghai Jiao Tong University School of Medicine, 1111 XianXia Road, Shanghai, 200050 China; 9 Department of Neurosurgery, Beijing Hospital, National Center of Gerontology, Beijing, China; 10 School of Computer and Information Technology, Beijing Jiaotong University, No. 3, Shangyuan Village, Haidian District, Beijing, 100089 China

**Keywords:** Cerebral small vessel disease, White matter hyperintensity, Functional network, Graph theoretical analysis, Cognitive impairment

## Abstract

**Background:**

White matter hyperintensity (WMH) is one of the typical neuroimaging manifestations of cerebral small vessel disease (CSVD), and the WMH correlates closely to cognitive impairment (CI). CSVD patients with WMH own altered topological properties of brain functional network, which is a possible mechanism that leads to CI. This study aims to identify differences in the characteristics of some brain functional network among patients with different grades of WMH and estimates the correlations between these different brain functional network characteristics and cognitive assessment scores.

**Methods:**

110 CSVD patients underwent 3.0 T Magnetic resonance imaging scans and neuropsychological cognitive assessments. WMH of each participant was graded on the basis of Fazekas grade scale and was divided into two groups: (A) WMH score of 1–2 points (n = 64), (B) WMH score of 3–6 points (n = 46). Topological indexes of brain functional network were analyzed using graph-theoretical method. T-test and Mann–Whitney U test was used to compare the differences in topological properties of brain functional network between groups. Partial correlation analysis was applied to explore the relationship between different topological properties of brain functional networks and overall cognitive function.

**Results:**

Patients with high WMH scores exhibited decreased clustering coefficient values, global and local network efficiency along with increased shortest path length on whole brain level as well as decreased nodal efficiency in some brain regions on nodal level (*p* < 0.05). Nodal efficiency in the left lingual gyrus was significantly positively correlated with patients' total Montreal Cognitive Assessment (MoCA) scores (*p* < 0.05). No significant difference was found between two groups on the aspect of total MoCA and Mini-mental State Examination (MMSE) scores (p > 0.05).

**Conclusion:**

Therefore, we come to conclusions that patients with high WMH scores showed less optimized small-world networks compared to patients with low WMH scores. Global and local network efficiency on the whole-brain level, as well as nodal efficiency in certain brain regions on the nodal level, can be viewed as markers to reflect the course of WMH.

**Supplementary Information:**

The online version contains supplementary material available at 10.1186/s12880-022-00769-7.

## Introduction

As the aging population increases, the incidence of cerebral small vessel disease (CSVD) is also rising. Statistically, CSVD accounts for approximately 10–30% [1] of ischemic stroke worldwide and is a major vascular contributor to cognitive impairment and dementia. White matter hyperintensity (WMH) is one of the typical neuroimaging manifestations of CSVD, and the prevalence rate of WMH in the Chinese population is as high as 70% [2–7]. Increasing shreds of evidence have shown that WMH correlates closely to CI (cognitive impairment) [8]. However, the underlying pathogenesis is not elucidated. Generally, there is no significant decline in cognitive function at early stages of WMH [9]. Cognitive decline tends to occur when the white matter is severely injured. Without early and timely intervention, some patients may experience further deterioration, moderate to severe cognitive impairment, and even subcortical vascular dementia [9]. Hence, understanding the characteristics of WMH at an early stage is of great significance. In recent years, resting functional magnetic resonance imaging (Rs-fMRI), as one of the main imaging techniques for constructing functional brain networks, has been widely applied to the research on the changes in brain function of WMH-related cognitive impairment [10]. Meanwhile, an analytical approach based on graph theory has been developed to explore the characteristics of the brain's structural and functional connectivity networks [11]. Relevant studies have confirmed that brain functional networks consist of brain regions that are highly correlated at the level of neural activity and partly reflect underlying structural connections with diffusion tensor imaging [12, 13]. To the best of our knowledge, several researchers have focused on the changes in structural network characteristics caused by white matter fibre bundle injury in WMH patients as well as their relationships with cognition [14–17]. However, few studies have investigated the topological properties of brain functional networks in patients with WMH. At the same time, the relationship between the severity of CSVD, as defined by WMH classification, and properties of brain functional networks has not been used frequently.

As a traditional MRI marker, WMH mainly reflects local brain injury [18–20]. While the construction of brain functional network focuses more on the integration of the whole brain information for functional changes caused by different structural injuries [20, 21], which is of great significance for the understanding of the occurrence and development mechanism of cognitive dysfunction in CSVD, meanwhile, understanding the differences of brain functional network characteristics in patients with different grades of WMH contributes to finding sensitive indicators that can reflect the course of the disease. Thus, in this paper we aim to identify the differences from the topology of brain functional network characteristics with different WMH gradings among CSVD patients. Furthermore, we explore the correlations between the different topology of brain functional network characteristics and cognitive assessment scores.

## Materials and methods

### Participants

In this study, we recruited 137 CSVD patients from a hospital. The inclusion criteria were as follows: (1) age 55 or older, (2) no history of brain trauma or dementia, (3) meet two or more of the following conditions: (1) moderate to extensive enlarged perivascular spaces in basal ganglia, (2) one or more asymptomatic lacune, (3) periventricular WMH Fazekas score 3 or deep WMH Fazekas score 2 or 3, 4)one or more deep cerebral microbleeds(CMBs), (4) bilateral hyperintensities visible on T2-weighted and FLAIR sequences located in the periventricular and deep white matter. The exclusion criteria were as follows: (1) A history of ischemic stroke with a diameter of > 15 mm or a history of cardiogenic cerebral infarction, (2) A history of hydrocephalus or brain tumor, (3) MRI contraindications, (4) leukodystrophy resulted from other causes (e.g. multiple sclerosis, etc.), (5) A history of diabetes.

### Data collection and neuropsychological evaluation

Throughout the study, we recorded in detail the demographic data of each subject, including age, gender, education level, clinical history of hypertension, hyperlipidemia, smoking and drinking history. Blood biochemical tests were performed on each subject, and the test results were tracked.

In addition, all subjects underwent multimodal MR scans. After that, the image quality of MR scan was checked and strictly checked by two radiologists. Eleven subjects with poor image quality were excluded.

To assess cognitive function, neuropsychological assessments were performed on each patient within a week of their MR examination. The Mini-mental State Examination (MMSE) as well as Montreal Cognitive Assessment (MoCA) scales were used to test the overall cognitive function of the patients and the total scores of the tests were recorded. Nine subjects who didn't cooperate well and seven subjects with missing test results were excluded. Finally, 110 subjects with complete data were included in the study.

This study was carried out in accordance with The Code of Ethics of the World Medical Association (Declaration of Helsinki) and was approved by the research ethics committee of Tongren Hospital, Shanghai Jiao Tong University School of Medicine. Each subject had signed an IRB informed consent form.

### WMH grading assessment

Two radiologists assessed the severity of the patient's WMH by observing fluid-attenuated inversion recovery (FLAIR) sequence images without knowledge of the subject's clinical information. On the basis of Fazekas grade scale [22], WMH in the patient's periventricular and deep white matter were graded separately, and the two grades were added together to record the total score (Table 1). Finally, WMH patients were divided into two groups: (A) WMH score of 1–2 points, (B) WMH score of 3–6 points. The evaluation of WMH scores in this study demonstrated excellent intra and inter reliability with a Kappa coefficient of 0.927 and Cronbach's α value of 0.892. Finally, we accepted the senior radiologist's evaluation result.

**Table 1:** Fazekas grade scale

Grade	Periventricular hyperintensity (PVH)	Deep white matter hyperintense signals (DWMH)
0	Absence	Absence
1	Caps or pencil-thin lining	Punctate foci
2	Smooth halo	Beginning confluence of foci
3	Irregular PVH extending into the deep white matter	Large confluent areas

### MRI acquisition

All participants were scanned by a Siemens 3.0 Tesla Skyra scanner (Siemens, Germany). A twenty-channel standard head coil with foam pads is used to limit head movement. 3D T1-weighted MPRAGE with TR/TE/TI = 2,400/2.13/1100 ms, FOV = 256*256mm^2^, voxel size = 1.0*1.0*1.0mm^3^, slice thickness = 1 mm and number of slices = 192. 3D T2W-FLAIR with: TR/TE/TI = 5000/395/1800 ms, FOV = 256*256mm2, voxel size = 1.0*1.0*1.0mm^3^, slice thickness = 1 mm and number of slices = 192. Rest-fMRI with: TR/TE = 2000/30 ms, FOV = 224*224mm^2^, voxel size = 3.5*3.5*4.0mm^3^, slice thickness = 4 mm and number of slices = 32. These slices cover the entire brain and the MRI data were evaluated by two radiologists who did not know the clinical information.

### Image processing

Resting-state fMRI data were pre-processed by using the SPM8 software package (http://www.fil.ion.ucl.ac.uk/spm/). First, the first ten slices were discarded without slice time correction and realignment to the first slice for head motion correction. Next, the obtained images were spatially normalized to the Montreal Neurological Institute space. Then, spatial smoothing was performed by using a resolution of 3*3*3 mm Gaussian kernel. Subsequently, filtering was performed using a bandpass filter (0.01–0.08 Hz), and linear trends were removed using linear regression method. Finally, multiple linear regression analysis was performed to remove the covariates which mainly included cephalic parameters, whole brain signals, white matter signals and cerebrospinal fluid signals.

### Network construction

Anatomical automatic labeling (AAL) atlas [23] was used to parcellate the whole brain into 90 functionally separate regions of interest (45 per hemisphere) in native space. Each region represents a node of the brain network. Then, GRETNA (https://www.nitrc.org/projects/gretna) was used to construct adjacency matrices of functional connectivity network. By computing pearson’s correlation coefficients between mean time series of each possible pair of the node for each subject [24], a 90 × 90 undirected correlation matrix was generated. To improve the normality of the correlation coefficients, a Fisher Z-transformation was used. To generate a binary undirected network, a set of sparse thresholds (ranging from 0.05 to 0.4, with steps of 0.05) were applied. The reason for choosing this range of sparsity thresholds is that networks were not fully connected at lower sparsity thresholds and were unlikely to maintain small-world architecture at higher sparsity thresholds [25].

In addition, we also constructed a weighted network, the steps of which are detailed in the Addirional file 1 (weighted network analysis results).

### Graph theory analysis

Five functional network parameters were calculated through Brain Connectivity Toolbox [26]. Small-world network properties related indexes were calculated including the average clustering coefficient (Cp), path length (Lp), the ratio of the Lp between real and random network (λ), the ratio of the Cp between real and random network (γ), and the ratio of γ to λ (σ). At whole brain level, we calculated global efficiency (Eglob) and local efficiency (Eloc). On nodal level, we calculated nodal efficiency (NodalE) and Eloc. The detailed descriptions of these metrics are given in Addirional file 1 (detailed descriptions of topological metrics).

### WMH volume measurement

In brief, the Wisconsin WMH Segmentation Toolbox19 (W2MHS, http://pages.cs.wisc.edu/~vamsi/w2mhs.html) was applied to automatically compute WMH volumes of each subject on FLAIR and 3D-T1 images.

### Statistical analysis

SPSS22.0 statistical software were applied for statistical analyses. We used t-test, chi-square test, and nonparametric test to compare the differences between the two sets of demographic, clinical characteristics, medical history, and neuropsychological data. Among them, age, some biochemical indexes and cognitive score of subjects are continuous variables with skewness distribution. Therefore, non-parametric test statistical method was adopted to compare the differences between the two groups. Some biochemical indexes of the two groups are continuous variables with normal distribution, so t test was adopted. Gender and some medical history are classified variables and chi-square tests were used. The topological indexes of brain network are continuous variables, some of which obey normal distribution, and some of which do not. When the topological attributes of brain network of two group subjects all follow normal distribution, the t-test was adopted to compare the differences between the two groups; when normal distribution is not followed, the Mann–Whitney U-test was adopted. We use t-test to analyze whether WMH volume and local efficiency are significantly related. To control class I errors, FDR correction is adopted in the multiple hypothesis testing. Then, binary linear regression analysis was applied for age correction. We used partial correlation analysis to evaluate relationship between different topological properties of brain functional networks and overall cognitive function. In partial correlation analysis, age, gender, and education level were considered as covariates. *p* < 0.05 was considered statistically significant.

## Results

### Demographic and clinical features

The demographic characteristics and clinical features of two groups are summarized in Table 2 [7]. No significant differences existed in terms of demographic data apart from the age (*p* < 0.05). The average age of subjects in Group A was significantly younger than that in Group B. For clinical data as well as neuropsychological data, there were no significant differences between two groups as well.

**Table 2:** Demographic, clinical characteristics and neuropsychological data [7]

Items	Group A (n = 64)	Group B (n = 46)	*p* value
Age	65 (7)	69 (13)	0.001**
Female, n (%)	44 (68.7)	33 (71.1)	0.736
Hypertension, n (%)	24 (37.5)	13 (31.7)	0.264
Hyperlipemia, n (%)	31 (56.4)	20 (57.1)	0.752
WMH volume	1.31 (1.94)	7.84 (7.08)	—
TC	4.52 (1.08)	4.38 ± 0.16	0.586
TG	1.21 (0.64)	1.50 (0.98)	0.199
HDL	1.37 ± 0.56	1.27 ± 0.69	0.140
LDL	2.85 ± 0.10	2.61 (1.42)	0.276
MMSE	29.00 (1.00)	29.00 (2.00)	0.091
MOCA	24.00 (5.00)	24.00 (5.00)	0.095

Values with normal distribution are presented as the mean ± stand deviation (SD); Values with non-normal distribution are presented as median (interquartile range)

^**^The difference between groups was statistically significant (*p* < 0.01)

This table in this manuscript has been published in J Stroke Cerebrovasc Dis, 2020, 29(12), 105,275. This table is a summary of demographic characteristics and clinical features of two groups. The current article included the same subjects and grouping criteria as previously published article. Therefore, the table has the same content. As previously published article that contains the this table in current article is part of our team's work as well. That is why this does not constitute dual publication

### Group differences in brain functional network characteristics

To explore the brain functional network characteristics differences in group A and group B, we extracted multiple features for the following analysis studies.

#### Efficiency-related global properties of brain functional networks with a significant difference between groups

**Fig. 1:**
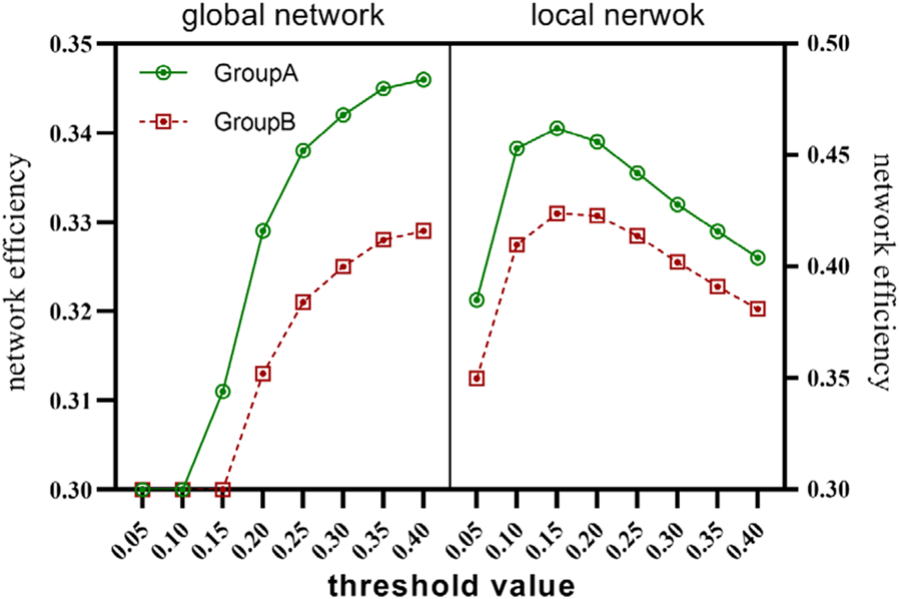
The global and local network efficiency of GroupA and GroupB. GroupA (group of patients with the 1–2 points WMH score, 64 cases). GroupB (group of patients with the 3–6 points WMH score, 46 cases). Two independent sample t test were used for statistical analysis

Making a general observation of small-world network index in both groups, we found that the the mean value of the ratio of Cp between real and random network $({\gamma }_{mean}, { \gamma }_{mean}^{GroupA}=1.99$ and${\gamma }_{mean}^{GroupB}=1.91$) was greater than 1, the mean value of the ratio of Lp between real and random network${( \lambda }_{mean}$, ${\lambda }_{mean}^{GroupA}=1.21$ and ${\lambda }_{mean}^{GroupB}=1.19$) was approximately equal to 1, and the mean value of the ratio of γ to λ (${\sigma }_{mean}$, ${\sigma }_{mean}^{GroupA}=1.59$ and ${\sigma }_{mean}^{GroupB}=1.55$) was greater than 1 under the different threshold value. According to the definition of small-world characteristic, if both satisfy γ > 1 and λ≈1, or σ = γ/λ > 1, then the network has small-world attribute. In addition, patients in group B showed lower Cp values (${C}_{p}^{groupB}=0.167$ and${L}_{p}^{groupB}=3.65$) and higher Lp values than those in group A (${C}_{p}^{groupA}=0.177$ and${L}_{p}^{groupA}=3.46$). The Cp (P = 0.048) values along with Lp (P = 0.009) values have a significant difference between groups (Table 3). Both Eglob and Eloc of patients in group B were significantly lower than that of patients in group A on the whole brain level (Fig. 1.). Significant differences also exist in Cp, Lp, Eglob and Eloc of brain weighted network between two groups (Additional file 1).

**Table 3:** Different global attributes of brain functional networks between groups

Attribute name	Threshold value	Group A	Group B	*p* vaule
	0.05	4.261	3.953	0.210
	0.1	2.585	2.453	
	0.15	2.013	1.937	
Gamma (γ)	0.20	1.705	1.654	
	0.25	1.508	1.467	
	0.3	1.376	1.346	
	0.35	1.283	1.26	
	0.4	1.214	1.194	
	0.05	1.541	1.513	
	0.1	1.276	1.233	
	0.15	1.196	1.172	
Lambda (λ)	0.20	1.165	1.149	0.049*
	0.25	1.152	1.138	
	0.3	1.144	1.132	
	0.35	1.14	1.129	
	0.4	1.136	1.125	
	0.05	2.828	2.71	
	0.1	2.055	2.008	
	0.15	1.692	1.659	
Sigma (σ)	0.20	1.466	1.442	0.184
	0.25	1.311	1.29	
	0.3	1.203	1.19	
	0.35	1.125	1.117	
	0.4	1.068	1.062	
	0.05	0.184	0.173	
	0.1	0.198	0.183	
	0.15	0.193	0.18	
Cp	0.20	0.184	0.173	0.048*
	0.25	0.175	0.166	
	0.3	0.168	0.16	
	0.35	0.162	0.155	
	0.4	0.157	0.151	
	0.05	5.82	6.1	
	0.1	3.742	3.862	
	0.15	3.246	3.411	
Lp	0.20	3.068	3.256	0.009**
	0.25	2.99	3.18	
	0.3	2.953	3.143	
	0.35	2.935	3.125	
	0.4	2.926	3.116	
	0.05	0.175	0.167	
	0.1	0.27	0.262	
	0.15	0.311	0.297	
Eglob	0.20	0.329	0.313	0.036*
	0.25	0.338	0.321	
	0.3	0.342	0.325	
	0.35	0.345	0.328	
	0.4	0.346	0.329	
	0.05	0.385	0.35	
	0.1	0.453	0.41	
	0.15	0.462	0.424	
Eloc	0.20	0.456	0.423	0.032*
	0.25	0.442	0.414	
	0.3	0.428	0.402	
	0.35	0.416	0.391	
	0.4	0.404	0.381	

^*^The difference between groups was statistically significant (0.01 < *p* < 0.05)

^**^The difference between groups was statistically significant (*p* < 0.01)

#### Different brain regions in efficiency-related local properties of brain functional networks between groups

**Fig. 2:**
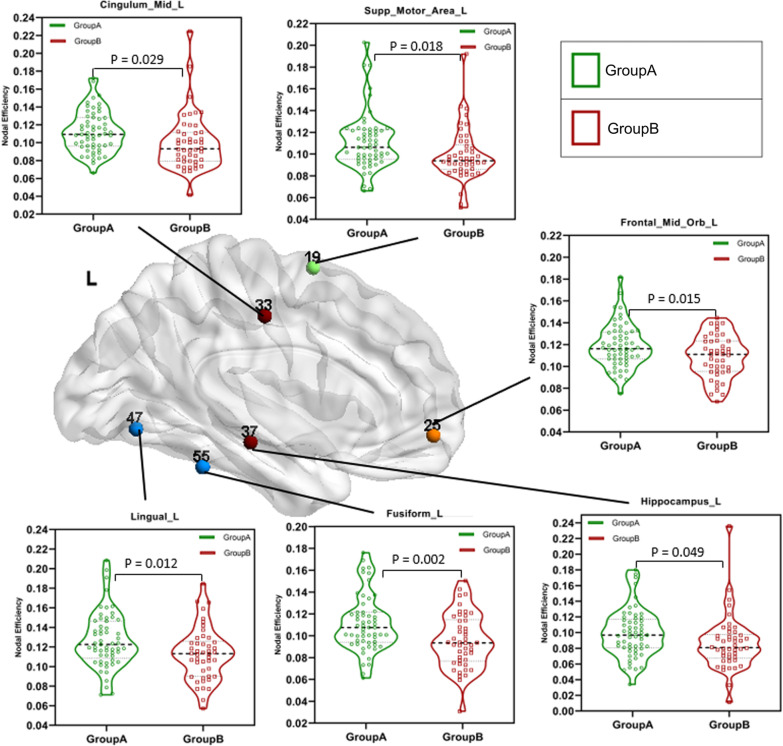
Results of alterations in the left-brain network node efficiency between group A and group B. The GroupA = group of patients with the 1–2 points WMH score. The GroupB = group of patients with the 3–6 points WMH score

**Fig. 3:**
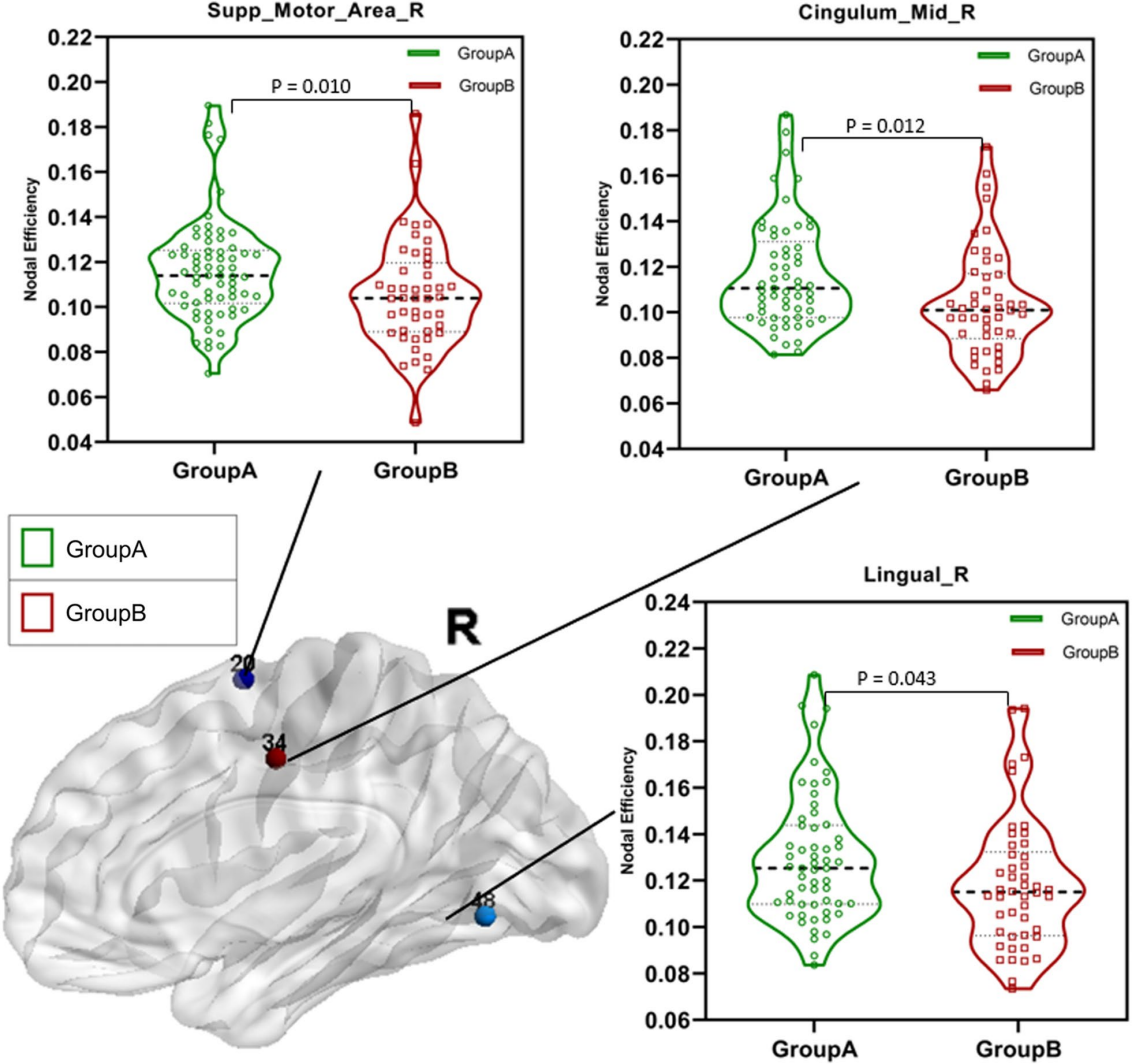
Results of alterations in the right-brain network node efficiency between group A and group B. The GroupA = group of patients with the 1–2 points WMH score. The GroupB = group of patients with the 3–6 points WMH score

This paper used the t-test to analyze the NodalE of patients in group A and group B at different brain network nodes (Table 4). The results showed that there was a significant difference in the NodalE between group A and group B in the bilateral supplementary motor area ($P\_L=0.018$ and $P\_R=0.010$), median cingulate and paracingulate gyri ($P\_L=0.029$ and $P\_R=0.012$) and lingual gyrus ($P\_L=0.012$ and $P\_R=0.043$), as well as left hippocampus($P\_L=0.015$), fusiform gyrus ($P\_L=0.043$) and medial orbital of superior frontal gyrus($P\_L=0.002$). And compared with patients in group A, patients in group B had significantly less NodalE in these brain network nodes (Fig. 2. and Fig. 3.), and the NodalE in group A is approximately 0.010 higher than the NodalE in group B. The results obtained by binary network analysis also exist in the weighted network analysis results (Additional file 1).

**Table 4:** Different brain regions in NodalE related local properties of brain functional networks between groups: coordinates are defined in standard space

Label	Brain regions	MNI coordinates			Mean value of NodalE		p-value
		X	Y	Z	GroupA	GroupB	(GroupA and GroupB)
19	Supp_Motor_Area_L	− 5	5	61	0.111	0.099	0.018*
20	Supp_Motor_Area_R	9	0	62	0.116	0.106	0.010**
25	Frontal_Mid_Orb_L	− 17	47	− 13	0.119	0.109	0.015*
33	Cingulum_Mid_L	− 5	− 15	42	0.112	0.100	0.029*
34	Cingulum_Mid_R	8	− 9	40	0.116	0.104	0.012*
37	Hippocampus_L	− 25	− 21	− 10	0.099	0.086	0.049*
47	Lingual_L	− 15	− 68	− 5	0.127	0.113	0.012*
48	Lingual_R	16	− 67	− 4	0.130	0.118	0.043*
55	Fusiform_L	− 31	− 40	− 20	0.111	0.095	0.002**

^*^The difference between groups was statistically significant (0.01 < *p* < 0.05)

^**^The difference between groups was statistically significant (*p* < 0.01)

NodalE: nodal efficiency

### Relationship between Eloc on nodal level and WMH volume

**Fig. 4:**
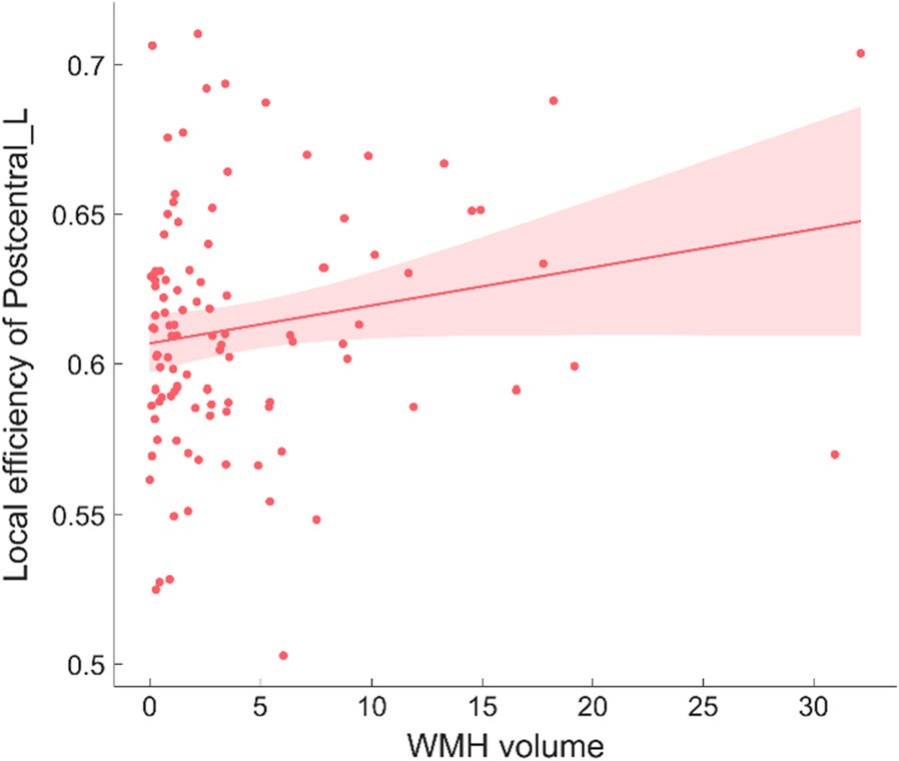
The correlation between WMH volume and the efficiency of Postcentral_L

In this paper, the correlation was analyzed between all the Eloc on the nodal level and the WMH volume (Table 5 and Fig. 4.), and we found that WMH volume is positively correlated with Eloc of the left postcentral gyrus (r = 0.203, *p* < 0.05).

**Table 5:** Correlation between Eloc on nodal level and the WMH volume

Brain regions	r	p
Postcentral_L	0.203	0.045*

^*^The difference between groups was statistically significant (0.01 < *p *< 0.05)

#### Correlation analysis between the different topological index of brain functional networks and cognitive function

Partial correlation analysis was used to find the relationship between the different topological index of brain functional networks and cognitive test scores (Table 6). Age, gender, and education level were considered as covariates*.* The results indicated that NodalE in the left lingual gyrus was significantly positively correlated with patients' total MoCA scores(r = 0.260, P = 0.026). No relationship was found between other different topological indexes of brain functional networks and total scores of MoCA as well as MMSE. The above results also appeared in the weighted network analysis results. Moreover, weighted network analysis showed a positive correlation between NodalE of the right superior temporal gyrus and MMSE score (Additional file 1).

**Table 6:** Correlation between NodalE in different brain regions and cognitive function

Brain regions	MoCA		MMSE	
	r	p	r	p
Lingual_L	0.260	0.026*	0.108	0.363

^*^The difference between groups was statistically significant (0.01 < *p* < 0.05)

## Discussion

In the present study, we investigated topological properties of resting state functional networks in CSVD subjects with different degree of WMH using graph theoretical analysis. The results of both binary network and weighted network analysis reveal that (i) the brain functional networks of all CSVD subjects own small-world properties, although the brain functional networks of subjects with low WMH scores are more optimized. Patients with high WMH scores show disturbed small-world networks (significant lower Cp and larger Lp), compared to a patient with low WMH scores. (ii) Compared to the patient with low WMH scores, a patient with high WMH scores display significant decreases in Eglob and Eloc on whole-brain level. (iii) Patients with high WMH scores exhibit decreased NodalE in cognition-related brain regions such as the superior frontal gyrus and hippocampus as well as other subregions. The analysis results of weighted network include all the analysis results of binary network. (iv) NodalE in the left lingual gyrus was positively correlate with patients' total MoCA score, which was found in both binary network and weighted network analysis. Besides, positive correlation also exists between NodalE of right superior temporal gyrus and patients’ MMSE score in analysis results of weighted network.

In line with previous studies, our finding indicates that small-world network properties are still conserved in patients with WMH of varying degrees of severity [27–29]. The human brain is considered as a complex but efficient neural network, which processes information efficiently with large Cp and short Lp [30, 31]. In our study, patients with high WMH scores show functional networks with significant lower Cp and larger Lp compared to a patient with low WMH scores. This suggests that patients with high WMH scores own a disturbed small-world network which might be less optimized than patients with low WMH scores. The Lp is an indicator of overall capacity for parallel information integration between remote brain regions [30, 31]. The Cp reflects the local connectivity and information transfer efficiency of the functional network [30, 32]. Therefore, increased Lp and decreased Cp indicate a weakened capacity for transmitting information between adjacent or distant cortical regions. This provides evidence for the explanation of cognitive decline.

On the aspect of brain functional network efficiency, an increasing number of studies have revealed that the Eloc and Eglob of brain networks decrease significantly in patients with subcortical ischemic vascular disease (SIVD) which is characterized by white matter lesions (WMLs) [28, 29, 33]. Our study indicates that the more severe WMLs is, the lower global and local network efficiency is on whole brain level. To investigate the reasoning, we hypothesize that as the severity of WMLs increases, the optimization degree of brain functional network in patients decreases. Lower Cp and longer Lp (as shown in our results above) imply a decrease in the efficiency of information transmission between both adjacent brain regions and remote brain regions. Considering the fact that Eglob is related to remote connections, thus reflecting the efficiency of information transmission between remote regions, and Eloc is mainly associated with short distance connections which indicates the ability to support specialized processing in densely connected brain regions [34], it is not difficult to understand that the Eloc and Eglob of brain functional networks with severe WMLs will be lower. Moreover, some researchers have suggested that alternate small-world network properties and reduced network efficiency in WMH patients attribute to disconnection between brain regions [35]. A considerable amount of research has shown that WMLs leads to the interruption of structural and functional connections between specific brain regions [36–40]. In theory, WMH patients with low Eg, Eloc, Cp and long Lp tend to suffer from cognition decline [28, 29, 41]. However, there is no significant difference in overall cognition between groups in our research. One explanation is that pre-clinical subjects enrolled in this study can tolerate certain degree of brain injury before the appearance of clinical symptoms. Compensatory connectivity theory states that in patients with CSVD, the brain maintains function by using an alternative network and increasing connections between parts of the brain regions [42–44]. Therefore,subjects enrolled in this study have not shown obvious cognitive dysfunction. There is no significant difference in cognitive function between two groups.

From the whole brain level, we learn the difference in cerebral functional network efficiency in patients with different severity of WMLs. Next, we need to recognize the specific brain areas that have significant changes in the efficiency with the progression of the CSVD. NodalE reflects the role of nodes in information processing [45, 46]. As network nodes are defined by functionally independent brain regions, NodalE also embodies the ability of communication and information transmission between neighbouring brain regions. Relevant studies have shown that WMLs lead to the destruction of the white matter pathway in the frontal-parietal lobe [33, 47]. In turn, the structural connectivity between these brain regions is disrupted. Structural separation also tends to have an impact on functional connectivity. Just as Sang discovered, patients with SIVD owned an alternative brain functional connectivity mainly involved orbitofrontal lobe, parietal lobe and temporal lobe [33]. All of these hinder the information transmission between different brain regions, which in turn leads to the decrease of NodalE. We hypothesize that as the severity of WMH increases, the communication between these brain regions becomes more difficult. With the increase of the severity of WMLs, the lower NodalE in certain brain regions will be observed. In addition, we notice that the reduction in NodalE in our study occurrs partly in the brain regions such as superior frontal gyrus (SFG) and hippocampus. Many kinds of researches have indicated that SFG is the vital brain region in charge of advanced cognitive function [48, 49], while the hippocampus plays an important role in the formation of episodic memory [50]. We speculate that WMH usually occurs in cognition-related brain regions. As WMH progresses, the connections between these brain regions are broken, thus reducing the efficiency of information transmission as well.

Besides, our results reveal the positive correlation between WMH volume and Eloc of Postcentral_L. The postcentral gyrus is the cortical area responsible for primary somatosensory. Outputs from postcentral gyrus project to hippocampus through parahippocampal cortex for further processing [51]. Eloc reflects the modularization degree and capability of information processing [52]. With the increase of WMH severity, the information processing capacity of the postcentral gyrus is improved instead, which is at odds with some previous research finding [53, 54]. In fact, the sample size of this study is small, and the correlation between WMH volume and Eloc of the left posterior central gyrus obtained by statistical analysis is weak (r = 0.203). Hence, further research needs to be done.

Further correlation analysis reveals that NodalE in the left lingual has a positive correlation with MoCA scores. Previous studies have demonstrated the close relationship between a disrupted topological organization of nodes and CI or dementia [55–57]. Meanwhile, a number of studies have confirmed that white matter microstructure injury is related to the decline of information processing capacity [58, 59]. NodalE has been confirmed to play the role of intermediary between white matter impairment and CI [41]. The lingual gyrus is primarily responsible for visual processing which may also be involved in logical analysis and visual memory processing [61]. With the significant increase of WMH load, NodalE in lingual gyrus decreases significantly. Thus, lingual gyrus cannot effectively integrate ongoing information with neighbouring brain regions. Therefore, cognitive function decreases with the decrease in the efficiency of lingual gyrus.

However, our study did not indicate the relationship between Eglob, Eloc as well as small-world attribute index of brain functional network and cognition on whole brain level. It is possible that when compared with the global attribute index, the relationship between node efficiency of specific brain regions and cognitive function of CSVD patients is more significant.

There are several limitations in current study. Firstly, this is a cross-sectional study. As the longitudinal data of the sample follow-up increases, we will gradually add the longitudinal analysis results. Secondly, we graded the patients with WMH by visual observation. This is somewhat subjective and cannot accurately reflect the severity of white matter lesions (WMLs). We invited two radiologists with senior experience to make grading calibration to minimize the deviation as much as possible. At the same time, we added the auto-segmented WMH volume to evaluate the WMH load. Thirdly, the CSVD is an age-related disease, all patients enrolled in our study were over 55 years old. While the WMH can be detected in 72–96% of the population over 60 years of age [61]. As a result, setting up an entirely healthy control group that matches the age of the patient group is difficult, resulting in the lack of a healthy control group. In the future, we will gradually expand the age range of the subjects.

## Conclusion

Small-world network properties are still conserved in patients with WMH of varying degrees of severity. While patients with high WMH scores show disturbed small-world networks which are less optimized compared to patients with low WMH scores. In addition, global efficiency and local efficiency on whole brain level as well as NodalE in certain brain regions on the nodal level can be viewed as markers to reflect the course of WMH.

## Supplementary Information

**Additional file 1.** Weighted network analysis results. Detailed descriptions of topological metrics

## Data Availability

The datasets generated and analysed during the current study are not publicly available as the relevant data of this experiment is still under study. The raw data can be available from the corresponding author (Jun Liu, E-mail: 1,554,165,538@qq.com) on reasonable request.
