# Supplementary material for: Impaired functional network properties contribute to white matter hyperintensity related cognitive decline in patients with cerebral small vessel disease
Source: BMC Med Imaging. 2022 Mar 9;22:40. doi: 10.1186/s12880-022-00769-7 (PMC8908649; doi:10.1186/s12880-022-00769-7)
Supplement: Supplementary file 1 — Additional file 1. Weighted network analysis results. Detailed descriptions of topological metrics [file 12880_2022_769_MOESM1_ESM.docx]

**Supplementary material**

1. **Detailed descriptions of topological metrics**
2. Path length (Lp) is the average of the shortest distance between all node pairs, and its calculation formula is as follows:

L_ij_=Min(path length from i to j), Lp=Mean(L_ij_).

1. Cp is the average value of clustering coefficient of each node. The clustering coefficient of node C(i) refers to the ratio of the actual number of edges E(i) in the subgraph Gi composed of Ki nodes connected to node i and the number of edges when the subgraph is fully connected. The calculation formula is as follows: $\text{C(i)}=\frac{\text{2E(i)}}{\text{k}_{\text{i}}\text{(k}_{\text{i}}-\text{1)}}$,.
2. Eglobal is the average reciprocal of the shortest paths between each pair of nodes, which can be computed as follows:

.

1. Ei-local is the average value of the shortest path of all nodes in the subgraph Gi, on which the average Elocal of the whole network can be obtained. The calculation formula is as follows:

,

(5) The nodal efficiency characterizes how efficient the parallel information transfer is when that node is in the network , which can be computed as follows:

$$E_{nodal}\left(i\right)=\frac{1}{N-1}\sum_{i\neq j\in G} \frac{1}{L_{ij}}$$

where N is the number of nodes in the network G, and Lij is the shortest path length between node i and j in the network.

1. **Weighted network analysis results**

The steps for weighted network construction are as follows: AAL brain region division template was used to divide the whole brain into 90 functionally separate regions of interest in order to define the network nodes. After constructing the functional connectome, pearson correlation coefficient between each ROI's averaged time courses was calculated to generate the correlation matrices (90 × 90). Fisher's r to Z transformation was applied to the corresponding correlation matrices. Finally, we extracted the upper triangle elements of the functional correlation matrices and obtained 90 × 89/2 = 4005 independent pairwise connectivity features.

An analytical approach based on graph theory was applied to analyze its topological properties. The results are as follow.

Table S1 Different global attributes of brain functional networks between groups

| attribute name | p-vaule |
| --- | --- |
| Cp | 0.007^**^ |
| Lp | ＜0.001** |
| Eglob | ＜0.001** |
| Eloc | 0.002** |

**：The difference between groups was statistically significant（p<0.01）

Cp: clustering coefficient; Lp: path length; Eglob: global network efficiency; Eloc: local network efficiency;

Table S2 Different brain regions in NodalE related local properties of brain functional networks between groups

| Label | Brain regions | p-vaule |
| --- | --- | --- |
| 1 | Precentral_L | 0.002** |
| 15 | Frontal_Inf_Orb_L | 0.009** |
| 19 | Supp_Motor_Area_L | 0.001** |
| 20 | Supp_Motor_Area_R | 0.002** |
| 25 | Frontal_Mid_Orb_L | 0.001** |
| 26 | Frontal_Mid_Orb_R | 0.009** |
| 31 | Cingulum_Ant_L | 0.009** |
| 33 | Cingulum_Mid_L | 0.001** |
| 34 | Cingulum_Mid_R | 0.001** |
| 37 | Hippocampus_L | 0.002** |
| 39 | Cingulum_Ant_L | 0.008** |
| 47 | Lingual_L | 0.004** |
| 48 | Lingual_R | 0.004** |
| 55 | Fusiform_L | 0.002** |
| 79 | Heschl_L | 0.007** |
| 81 | Temporal_Sup_L | 0.004** |
| 83 | Temporal_Pole_Sup_L | 0.004** |
| 84 | Temporal_Pole_Sup_R | 0.001** |
| 88 | Temporal_Pole_Mid_R | 0.003** |

**：The difference between groups was statistically significant（p<0.01）

NodalE: nodal efficiency

Table S3 Correlation between NodalE in different brain regions and cognitive function

| Brain regions | MoCA | | MMSE | |
| --- | --- | --- | --- | --- |
|  | r | p | r | p |
| Lingual_L | 0.218 | 0.036* | 0.106 | 0.418 |
| Temporal_Pole_Sup_R | 0.152 | 0.243 | 0.106 | 0.016* |

*: The difference between groups was statistically significant（0.01<p<0.05）
